# The impact of the flipped classroom model on college students' physical exercise behavior: a chain mediation effect of self-directed learning ability and exercise motivation

**DOI:** 10.3389/fpubh.2026.1804884

**Published:** 2026-07-13

**Authors:** Hechong Yang, Shuaichao Cheng, Guangxin Cheng

**Affiliations:** 1 School of Physical Education, Shandong University of Technology, Zibo, China; 2 School of Physical Education, Shaoyang University, Shaoyang, China; 3 School of Sports, Southwest University, Chongqing, China

**Keywords:** chained mediation, flipped classroom, intrinsic motivation, physical exercise behavior, self-directed learning ability, university public physical education

## Abstract

**Background:**

Encouraging consistent physical exercise habits among college students is a significant concern in public health. University physical education courses offer advantages in shaping healthy practices, yet conventional teaching often fails to convert classroom learning into lasting exercise routines. This study investigates the impact of Flipped Classroom (FC) model on students' exercise behavior and tests the chained mediation of self-directed learning (SDL) ability and autonomous motivation (AM). We hypothesize that FC positively influences exercise behavior through the sequential mediation of SDL ability and AM.

**Methods:**

This quasi-experimental study used a pre-post design in a Chinese university public physical education course. Six naturally occurring classes were randomly allocated to FC (*n* = 3) or traditional (*n* = 3) groups. A total of 308 students completed baseline (T1) and post-intervention (T2) assessments. Physical exercise behavior was measured with the International Physical Activity Questionnaire Short Form. Psychological characteristics were assessed using the Self-Directed Learning Ability Scale and the Behavioral Regulation in Exercise Questionnaire-3. Statistical analyses were performed using SPSS 26.0 and Mplus 8. Linear mixed models examined primary instructional effects. Structural equation modeling and bias-corrected bootstrap tested the sequential mediation pathway.

**Results:**

After adjusting for baseline baseline physical activity (PA), gender, body mass index, and class clustering, FC group showed significantly greater overall physical activity and moderate-to-vigorous physical activity (MVPA) than the control group. The likelihood of meeting physical activity recommendations was markedly higher in FC group. The structural equation model fit the data well. Both SDL ability and AM served as significant mediators, with a notable chained mediation pathway.

**Conclusion:**

FC model effectively fosters short-term physical exercise behavior in college students by enhancing SDL ability and reinforcing AM, as measured immediately post-intervention. These findings suggest promising educational and public health benefits, though they represent immediate post-course compliance rather than confirmed long-term habit formation, warranting future longitudinal research.

## 1 Introduction

Consistent physical exercise is essential for the physical and mental health of adolescents (1, 2). The university phase marks a transition from “managed” to “self-managed” health behaviors, offering an opportunity to cultivate lasting healthy habits (3, 4). However, many college students face barriers such as academic pressure, sedentary behavior, and insufficient physical activity support (5, 6). University public physical education courses, due to their wide reach and institutional structure, serve as a key platform for influencing exercise habits (7, 8).

Conventional PE teaching relies heavily on instructor-led lectures and standardized drills (9, 10). Limited class time, difficulty addressing individual differences, and weak post-class reinforcement create a disconnect between classroom learning and extracurricular practice (11). This “structural contradiction” primarily reflects a failure of motivational transfer rather than skill retention: students may acquire motor skills in class but lack the autonomous motivation and self-regulatory strategies to sustain exercise independently after the course ends. FC model is an instructional innovation designed to address this gap (12–14). FC shifts basic learning—such as information acquisition and movement demonstrations—to online pre-class activities (15), freeing in-class time for interaction, error correction, and practical application (16). This creates a continuous learning cycle across pre-class, in-class, and post-class phases. In PE, this approach may prolong learning duration, strengthen post-class consolidation, and encourage students to apply classroom knowledge to daily exercise (17–19). Research indicates that FC positively influences psychological outcomes such as intrinsic motivation, self-efficacy, and learning satisfaction (20, 21). However, most studies focus on classroom learning effectiveness, neglecting physical exercise behavior—particularly extracurricular participation and persistence—which has greater public health relevance (22, 23).

In FC, SDL ability may serve as a critical link between instructional model and exercise behavior (24–26). FC demands substantial pre-class preparation and self-regulation (27), and students' SDL abilities affect implementation quality (28). SDL involves goal setting, strategy implementation, self-monitoring, and resource allocation. Importantly, academic SDL (learning a motor skill) and exercise SDL (managing a personal workout routine) share core self-regulatory processes: goal-setting, self-monitoring, and adaptive strategy use. FC model trains these transferable skills by requiring students to plan pre-class viewing, self-assess via quizzes, monitor post-class practice through video uploads, and reflect via journals. These activities build metacognitive infrastructure for lifelong physical activity self-management. Improved SDL ability enhances mastery and competence, core elements of self-determination theory (29–32), leading to more autonomous exercise motivation and sustained behavior (33).

Despite theoretical support, empirical research systematically testing whether FC's impact on exercise behavior is sequentially mediated by SDL ability and AM remains limited. This study develops and tests a chained mediation model: FC → SDL ability → AM → physical exercise behavior. Specifically, we aim to determine (1) whether FC positively affects college students' physical exercise behavior, (2) whether SDL ability and AM serve as mediators, and (3) whether these two factors form a chained mediation pathway (34–36).

**Figure 1:**
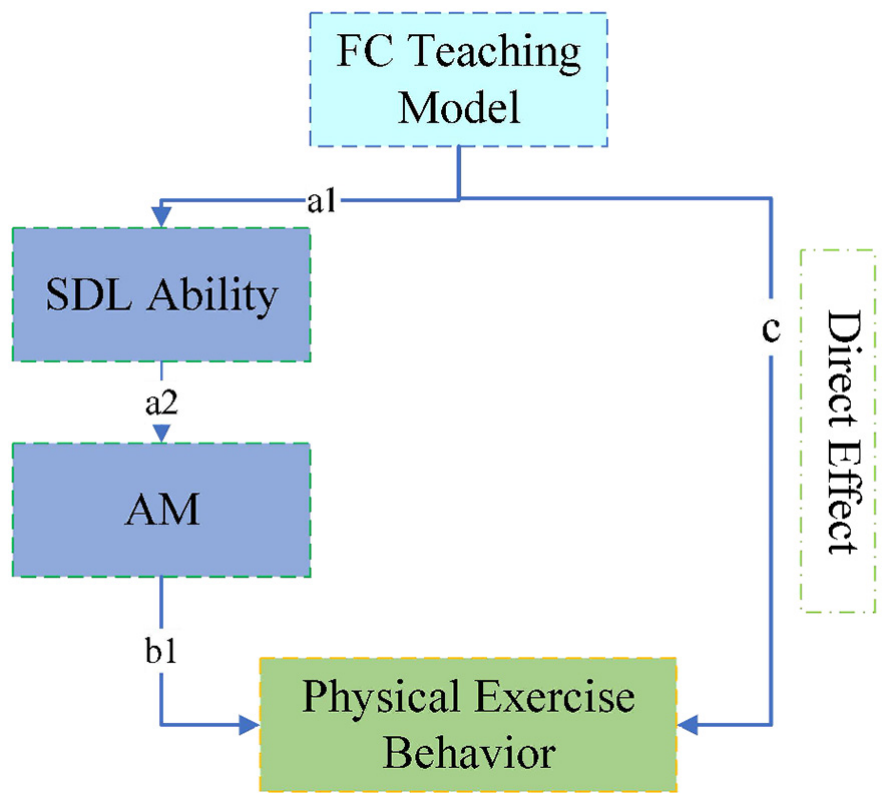
Proposed chained mediation model of FC teaching model on college students' physical exercise behavior, mediated by SDL ability and AM. Path a1, a2, b1, and c' denote the hypothesized relationships.

Figure 1 presents the proposed chained mediation model. Path a1 represents the effect of FC on SDL ability; path a2 represents the effect of SDL ability on AM; path b1 represents the effect of AM on physical exercise behavior; path c' represents the direct effect of FC on physical exercise behavior after controlling for mediators.

## 2 Methods

### 2.1 Research design

This study used a two-arm, cluster quasi-experimental pretest-posttest design to investigate the effect of FC teaching approach on college students' physical exercise behavior and the mediating roles of SDL ability and AM (37–39). Due to practical scheduling constraints, existing classes were used to form groups: three naturally occurring classes in the experimental group received FC instruction, and three naturally occurring classes in the control group received traditional instruction. The six classes were randomly allocated to condition by the research team using a simple randomization procedure (e.g., coin flip), conducted by the lead researcher who was not involved in instruction (40–42); allocation concealment was maintained until intervention start. Data were collected at two time points: T1 (baseline, week 1) and T2 (posttest, week 16).

The primary outcome was physical exercise behavior. SDL ability and AM were examined as mediators. Analyses adjusted for T1 baseline levels of exercise, gender, and body mass index (BMI). Robustness checks included alternative outcome metrics and sensitivity analyses.

### 2.2 Research context and participants

The study was conducted at Shandong University of Technology (Shandong Province, China) within mandatory public PE courses for first- and second-year undergraduates. This university was selected because it had the digital infrastructure (Lingtong/Yuketang platforms) to support FC and instructors had prior blended learning experience, ensuring intervention fidelity. Both groups followed the same syllabus and grading criteria (60% skill demonstration, 40% attendance/participation). The same two instructors taught both experimental and control groups (each instructor taught one FC class and one traditional class), controlling for instructor characteristics (43, 44).

Convenience and cluster sampling were used: six classes (three per condition) from the same semester were enrolled (45–48). Inclusion criteria were undergraduate students aged 18–24 enrolled in regular PE courses, free from serious illness, and providing informed consent. Exclusion criteria included severe injury/illness during the study, absences exceeding 20% of total class hours (threshold based on institutional attendance policy to ensure adequate intervention dose), invalid questionnaire responses, or inability to match T1 and T2 data (Table 1).

**Table 1:** Inclusion and exclusion criteria.

Inclusion criteria	Exclusion criteria
Undergraduate students aged 18–24; currently enrolled in regular public physical education courses for the semester and expected to complete the entire semester of study; free from serious illness or explicit medical restrictions on participation in physical activities; voluntarily participating in the study and having signed an informed consent form	Severe sports injuries or illnesses occurring during the study period that prevent continued course participation; absences exceeding 20% of total class hours (or the minimum attendance requirement for course assessment as stipulated by the institution); invalid questionnaire responses (e.g., extremely short completion time, homogeneous responses, failure to pass attention detection questions, missing key variables >20%); inability to correctly match T1 and T2 through anonymous coding

The target sample size of 308 was determined by class enrollment numbers, accounting for 10–20% attrition, which meets recommended minimums for structural equation modeling (SEM) and bootstrap mediation testing (≥300). A *post-hoc* sensitivity analysis showed 80% power to detect a small-to-medium indirect effect (β ≥ 0.05) at α = 0.05. For cluster-level effects (six classes, average intraclass correlation ICC = 0.02), the design effect was 1 + (average cluster size – 1) × ICC = 1 + (51.3 – 1) × 0.02 ≈ 2.0, resulting in an effective sample size of approximately 154, which remains adequate for primary analyses.

### 2.3 Intervention

The experimental group received a 16-week FC intervention (one 90-min class per week) structured around a “pre-class, in-class, post-class” framework (detailed in Table 2). Pre-class: students accessed micro-lectures, task sheets, and online quizzes via Learning Pass/Yu Classroom platforms. In-class: activities focused on practice-feedback-repractice cycles, group work, and individualized feedback. Post-class: students completed exercise check-ins, video uploads, reflection logs, and received supplementary resources (49–52). The control group followed traditional instruction with in-class lectures and practice, no systematic pre-class online learning, and no structured post-class assignments. Both groups followed the same content and pacing. Implementation fidelity was ensured through an FC manual, instructor training, weekly checklists, and prevention of information contamination between groups (53, 54).

**Table 2:** Instructional content for experimental and control groups.

Experimental group (FC teaching model)			Control group
Pre-class phase: online self-directed learning	Mid-lesson phase: high-density practice and feedback	Post-lesson phase: consolidation and behavioral transfer	Regular instruction
Task packages are pushed via the platform 48 hours before each class: 1. Micro-lessons and movement demonstration videos: 2–4 segments per class, each lasting 5–10 min, covering key movement points, common mistakes, correction tips, and safety essentials; 2. Learning task sheets: Clearly defining learning objectives, critical technical points, self-assessment questions, and practice considerations; 3. Online quiz: 5–10 questions (multiple choice/true/false/short answer) to assess comprehension and guide focus; 4. Pre-class feedback: Submit questions or self-assessments. Instructors use backend data (completion rates, quiz scores, common errors) for in-class diagnostics, enabling “teaching based on learning.”	Classroom instruction centers on the “practice-feedback-repractice” cycle: 1. Brief pre-class Q&A addressing common errors, with demonstrations of key corrective movements; 2. Group practice and peer assessment (using action evaluation rubrics) to enhance practice intensity and engagement; 3. Teacher circulation guidance with individualized feedback, emphasizing movement quality and error correction strategies; 4. Incorporate group tasks or contextualized practice (e.g., skill combinations, challenge activities, simplified competitions) to boost mastery experiences and engagement; 5. Conclude with key point summaries, assign post-class reinforcement tasks, and outline next-session preparation requirements.	After class, use the platform to facilitate the transition from “classroom learning → extracurricular practice”: 1. Exercise check-ins: 2–3 times per week, recording exercise type, duration, and intensity (subjective RPE or light/moderate/high); 2. Video Uploads: Submit practice videos (single-movement clips/combination drills) per assignment requirements; instructors or teaching assistants provide brief feedback; 3. Reflection Logs: Dedicate 1–2 min post-class to reflect (key takeaways, challenges, next goals) to foster self-monitoring; 4. Resource Supplementation & Q&A: Distribute supplementary correction videos addressing common issues and conduct online Q&A sessions.	The control group followed the school's standard public physical education curriculum: instructors provided explanations and demonstrations, students practiced in class, and received feedback on corrections during lessons. This group did not engage in systematic pre-class micro-lesson learning or online quizzes. Post-class activities did not require unified check-ins or video uploads; students only completed necessary exercises as per regular course requirements. Both groups aligned as closely as possible in teaching content and pacing to minimize the impact of content differences on outcomes.

### 2.4 Measures

Physical exercise behavior was assessed using the International Physical Activity Questionnaire Short Form (IPAQ-SF), providing continuous total PA (MET-min/week) and moderate-to-vigorous PA (MVPA, min/week), as well as a binary indicator of meeting PA recommendations (≥150 min/week MVPA). SDL ability was measured using the Self-Directed Learning Readiness Scale (58 items, 5-point Likert). Autonomous motivation was derived from the Behavioral Regulation in Exercise Questionnaire-3 (BREQ-3), calculated as the mean of intrinsic, integrated, and identified regulation subscales. All scales demonstrated acceptable reliability (Cronbach's α > 0.80) and validity (CFA fit indices met criteria). Covariates included age, gender, BMI, and baseline PA (55–58).

### 2.5 Data collection and quality assurance

Data were collected in classroom settings at T1 and T2 by trained researchers. Anonymity was emphasized, and instructors were absent during questionnaire completion to reduce social desirability bias. Anonymous coding enabled matching of pre- and post-test data. Invalid responses (e.g., extremely short completion time, missing >20% data, homogeneous answers) were excluded (59–61). Extreme outliers in PA data were truncated following IPAQ guidelines. Missing data (< 5%) were handled using full information maximum likelihood. Process data (platform engagement metrics) were used for fidelity checks but not included as mediators.

### 2.6 Statistical analysis

All analyses were performed using SPSS 26.0 and Mplus 8, with α = 0.05 (two-tailed). Descriptive statistics and baseline equivalence were examined using independent *t*-tests and chi-square tests. Confirmatory factor analysis (CFA) assessed measurement model fit for SDLRS and BREQ-3. The primary effect of FC on PA outcomes was examined using linear mixed models (LMMs) for continuous outcomes and generalized linear mixed models (GLMMs) for the binary outcome, with class as a random intercept and covariates (T1 PA, gender, BMI). The chained mediation model (teaching mode → SDL ability → AM → PA) was tested using SEM with bias-corrected bootstrap (5,000 resamples) to estimate indirect effects and 95% confidence intervals (62–66) (Table 3).

**Table 3:** Overview of research variables, measurement instruments, and operationalization methods.

Variable categories	Variable name	Measuring tools	Number of items	Dimension/composition	Scoring method	Variable calculation method	Reliability and validity testing
Independent variable	Teaching model	Grouping variable	–	FC/traditional classroom	Categorical variable	0 = control group; 1 = experimental group	–
Mediating variable 1	SDL ability	Self-directed learning readiness scale (SDLRS)	58	Multi-dimensional structure (primarily based on overall capability)	Likert 5-point rating scale; reverse-scored for reverse-scored items	SDL = 58-item mean (or total score); higher scores indicate stronger SDL ability	Cronbach's α, McDonald's ω; confirmatory factor analysis (CFA) (CFI, TLI, RMSEA, SRMR); CR, AVE
Mediating variable 2	AM for exercise	Behavioral regulation in exercise questionnaire-3(BREQ-3)	24	Internal, integrated, identification, internalization, external, unmotivated	Likert 5-point scale; mean scores for each dimension	AM = (intrinsic + identified + integrated)/3; supplement: controlled motivation, no motivation	Scales *α, ω*; six-factor CFA; CR, AVE; discrimination validity (HTMT)
Dependent variable	Physical exercise behavior (continuous)	International physical activity questionnaire–short form (IPAQ-SF)	7	High-intensity, moderate-intensity, walking, sedentary	Frequency (days/week) × duration (minutes/day)	Total PA = sum of MET-min/week across all intensities; MVPA = moderate-to-vigorous intensity minutes/week	Abnormal value handling according to IPAQ guidelines; descriptive and structural consistency tests
Dependent variable	Physical exercise behavior (classification)	IPAQ-SF derived indicators	–	Meets standards/does not meet standards	Binary classification	Whether the recommended level is met (e.g., ≥150 min/week of moderate-intensity or equivalent MVPA)	Consistency test with continuous indicators
Covariate	Demographic variables	Self-designed questionnaire	Several	Gender, age, grade level	Classification/continuous	Gender (0/1), age, grade level	Descriptive statistics
Covariate	BMI	Self-reported height and weight	2	Weight, height	Continuous	BMI = kg/m^2^	Descriptive statistics
Covariate	Baseline exercise level	IPAQ-SF(T1)	7	Weight, height	Continuous	T1 total PA or T1 MVPA	Enter the model as a covariate

Terminology note: Throughout this manuscript, AM refers to the self-determined, internally regulated motivation assessed by the BREQ-3 (intrinsic, integrated, and identified subscales). “Exercise motivation” is used only as a general descriptor; the specific construct is consistently labeled as AM.

### 2.7 Ethical considerations

The study complied with the Declaration of Helsinki and was approved by the Ethics Committee of Shandong University of Technology (approval No.: SDUT20250828). All participants provided written informed consent and were assured that participation or withdrawal would not affect course grades.

## 3 Results

### 3.1 Sample characteristics

Of 338 initial participants, 308 valid cases (91.1%) were included after matching and cleaning. The sample comprised 51.4% males, mean age 19.82 years. No significant demographic differences existed between experimental (*n* = 171) and control (*n* = 167) groups at baseline.

### 3.2 Baseline equivalence and descriptive statistics

As shown in Table 4, groups did not differ significantly in T1 SDL ability, AM, total PA, MVPA, or PA guideline attainment (all *p* > 0.05). Table 5 shows that at T2, the experimental group had higher SDL ability, AM, and PA compared to T1, while controls showed minimal change. Correlations among T2 variables supported the hypothesized sequence (SDL-AM *r* = 0.48, AM-PA *r* = 0.41; *p* < 0.001).

**Table 4:** Baseline characteristics and equivalence between groups (T1).

Variable	Experimental group (*n* = 171)	Control group (*n* = 167)	*t*/*χ^2^*	*p*
Age (years)	19.84 ± 1.02	19.79 ± 1.06	0.42	0.67
Sex (male, %)	52.6	50.3	0.18	0.67
BMI (kg/m^2^)	22.31 ± 2.84	22.18 ± 2.76	0.41	0.68
SDL	3.42 ± 0.41	3.39 ± 0.44	0.61	0.54
AM	3.51 ± 0.56	3.48 ± 0.59	0.48	0.63
Total PA (MET-min/week)	1826 ± 764	1769 ± 781	0.73	0.47
MVPA (min/week)	143 ± 68	139 ± 71	0.56	0.58
Meeting PA guidelines (%)	41.5	39.8	0.10	0.75

Values are presented as mean ± SD or percentage. Group differences were examined using independent-samples *t* tests or chi-square tests as appropriate.

**Table 5:** Descriptive statistics and correlations among main variables (T1 and T2).

Variable	M ±SD (T1)	M ±SD (T2)	1	2	3
SDL ability	3.41 ± 0.43	3.68 ± 0.46	–		
AM	3.50 ± 0.58	3.77 ± 0.61	0.48^*^	–	
Total PA (MET-min/week)	1799 ± 773	2146 ± 821	0.29^*^	0.41^*^	–

Pearson correlation coefficients are reported using T2 data. ^*^p < 0.001.

### 3.3 Descriptive statistics and correlation analysis

Table 5 delineates the descriptive statistics for essential factors at T1 and T2. In comparison to T1, the experimental group displayed increases in SDL ability, AM, and physical exercise behavior at T2, but the control group showed negligible change throughout the same timeframe. Correlation analysis indicated that at T2, SDL ability exhibited a substantial positive correlation with AM (*r* = 0.48, *p* < 0.001). AM had a substantial positive correlation with physical exercise behaviors (Total PA, MVPA) (*r* = 0.41, *p* < 0.001). A notable positive association was identified between SDL ability and physical exercise behavior, but its strength was marginally weaker than that between ability and motivation. This correlational framework corresponds with the suggested “ability-motivation-behavior” progressive process in the research hypothesis.

### 3.4 Main effects of FC on physical activity

Table 6 presents the main effects. After adjusting for covariates and class clustering, FC significantly increased total PA (β = 0.27, 95% CI [0.11, 0.43], *p* = 0.001) and MVPA (β = 0.23, 95% CI [0.09, 0.37], *p* = 0.002). The odds of meeting PA guidelines were 1.87 times higher in FC group than control (95% CI [1.21, 2.89], *p* = 0.004).

**Table 6:** Main effects of instructional condition on physical exercise behavior (T2).

Outcome variable	β/OR	SE	95% CI	*p*
Total PA (MET-min/week)	β = 0.27	0.08	[0.11, 0.43]	0.001
MVPA (min/week)	β = 0.23	0.07	[0.09, 0.37]	0.002
Meeting PA guidelines	OR = 1.87	–	[1.21, 2.89]	0.004

Models were adjusted for baseline physical activity, sex, BMI, and class-level clustering. Continuous outcomes were analyzed using LMMs; categorical outcome was analyzed using a generalized linear mixed model.

### 3.5 Chained mediation model

SEM results supported the hypothesized chained mediation: total effect of FC on PA = 0.31 (95% CI [0.18, 0.45]); direct effect after including mediators = 0.14 (95% CI [0.02, 0.26]). The indirect effect via SDL ability alone was 0.07 (95% CI [0.03, 0.13]), via AM alone was 0.06 (95% CI [0.02, 0.11]), and the chained indirect effect via SDL ability then AM was 0.04 (95% CI [0.01, 0.08]). All bootstrap CIs excluded zero, indicating statistically significant partial mediation.

## 4 Discussion

This quasi-experimental study found that the FC model significantly improved college students' physical activity levels compared to traditional instruction, with effects observed in both continuous PA and guideline attainment. Furthermore, the relationship was sequentially mediated by SDL ability and AM, supporting the hypothesized “ability–motivation–behavior” chain. These findings extend the literature by elucidating the psychological mechanisms through which an instructional innovation translates into health-relevant behavioral outcomes.

### 4.1 Interpretation of main findings

The observed effect size (β = 0.27 for total PA, OR = 1.87 for meeting guidelines) is modest in magnitude but carries meaningful public health implications. Even a 0.27 standardized increase in PA, if sustained at the population level, could contribute to reducing sedentary lifestyle-related chronic diseases among young adults (3). The odds ratio of 1.87 indicates that FC students were nearly twice as likely to achieve recommended activity levels—a clinically relevant threshold.

Several mechanisms may explain why FC facilitates behavioral spillover from in-class learning to extracurricular activity (47). By restructuring the learning cycle across pre-class, in-class, and post-class phases, FC extends the temporal boundaries of PE beyond the classroom, creating multiple touchpoints for behavioral reinforcement. This continuous engagement may reduce the “fade-out” effect commonly observed in traditional PE. Additionally, the FC model's emphasis on self-paced pre-class preparation and post-class reflection may lower the perceived barrier to initiating exercise outside class, as students develop greater familiarity with movement techniques and self-monitoring routines (32). This finding aligns with prior meta-analyses on FC in health education (17, 24) but extends evidence to the specific context of university PE—a setting where sustained behavioral transfer has historically been elusive. Unlike previous studies that focused primarily on knowledge acquisition or skill proficiency (13, 23), our results demonstrate that FC can influence behavioral outcomes with direct public health relevance. This distinction is critical because skill acquisition without behavioral enactment yields limited population health benefits.

### 4.2 Mediation pathways

SDL ability emerged as a significant mediator of the FC–PA relationship, consistent with theoretical accounts that FC enhances self-regulatory skills (33). This finding is theoretically coherent: FC's instructional design explicitly trains students to plan, monitor, and evaluate their learning—processes structurally analogous to managing an independent exercise routine. When students internalize these competencies in an academic context, they can transfer them to health behaviors. Moreover, SDL ability predicted AM, which in turn predicted PA. This sequential pathway suggests a temporal and psychological ordering: FC first builds competence (through mastery of self-regulation), which satisfies the need for competence—a core psychological need in Self-Determination Theory—and this competence satisfaction fosters autonomous motivation for exercise. The relative magnitude of the indirect effects is worth noting: the indirect effect via SDL ability alone (0.07) was slightly larger than that via AM alone (0.06), while the chained indirect effect (0.04) was smaller but still statistically significant. This pattern suggests that SDL ability plays a primary mediating role, while AM serves as a secondary but important amplifying factor. The chained pathway, though modest, is theoretically significant because it captures the sequential process from pedagogical input to cognitive skill development to motivational internalization to behavioral output.

### 4.3 Theoretical contributions and practical implications

This study contributes to the literature in three principal ways. First, it shifts FC research focus from classroom-centric outcomes to public health-relevant exercise behavior—a domain with broader societal implications. Second, it empirically tests a chained mediation model integrating self-regulated learning theory and self-determination theory, demonstrating that cognitive and motivational mechanisms operate sequentially. Third, it provides evidence that pedagogical innovations can have measurable “spillover effects” beyond the immediate educational context. Practically, FC offers a viable strategy for PE reform, but its scalability requires careful consideration of resource demands. Implementing FC requires substantial instructor time for video production, platform management, and online feedback—resources that may be scarce in lower-resourced universities. Our findings suggest that the most critical active ingredient may be the structured self-regulation opportunities (e.g., quizzes, video uploads, reflection logs) rather than the specific technological platform. Therefore, simpler, lower-cost platforms (e.g., WeChat groups, open-source LMS) could potentially achieve similar effects if they support core self-regulatory processes. Additionally, teacher education programs should incorporate competencies in fostering self-regulated learning and providing individualized feedback to prepare instructors for FC-based PE instruction.

### 4.4 Limitations

Several limitations warrant caution. First, the quasi-experimental design precludes definitive causal inference; unmeasured confounding may remain. The absence of individual-level randomization means selection bias cannot be entirely ruled out; cluster-randomized trials would provide stronger causal evidence. Second, self-reported PA is susceptible to recall bias and social desirability, and the FC's high-engagement environment may amplify a “halo effect” (students overreporting to please instructors). While we mitigated this by ensuring instructor absence and emphasizing anonymity, differential attentional investment may have fostered subconscious overreporting. Future studies should incorporate objective activity monitors to validate self-reported levels (42). Third, the same instructors taught both conditions, which controls for instructor effects but may introduce cross-contamination. Using fully crossed or independent instructor designs in future studies would further isolate the teaching model effect. Fourth, the sample was drawn from a single university, limiting generalizability to other institutional and cultural contexts. Fifth, and most critically, T2 measurements occurred immediately post-course (week 16); results reflect short-term compliance rather than sustained habit change. Longitudinal follow-up is essential.

### 4.5 Future directions

Future research should pursue several directions: implement cluster-randomized controlled trials with extended follow-up (6–24 months post-intervention) to establish causality and assess long-term maintenance; incorporate objective activity monitors (accelerometers, smartwatches) to validate self-reported PA; test FC adaptations across diverse institutional and cultural contexts to examine generalizability;investigate potential moderators (e.g., baseline SDL ability, digital literacy, prior exercise habits) to identify subgroups that benefit most; examine comparative effectiveness of different FC components (video format, quiz frequency, feedback modalities) to identify active ingredients and optimize intervention efficiency; and evaluate cost-effectiveness of scalable FC models, comparing high-tech and low-tech implementations to inform resource allocation.

## 5 Conclusion

This study provides evidence that the flipped classroom model enhances short-term physical exercise behavior in college students through a sequential chain mediation of self-directed learning ability and autonomous motivation. While the findings are promising for public health pedagogy, they represent immediate post-course compliance, not confirmed long-term habit formation. Replication in diverse settings with objective measures and extended follow-up is necessary before concluding that FC produces sustainable behavior change.

## Data Availability

The original contributions presented in the study are included in the article/supplementary material, further inquiries can be directed to the corresponding author.
